# Giving Up on Consciousness as the Ghost in the Machine

**DOI:** 10.3389/fpsyg.2021.571460

**Published:** 2021-04-30

**Authors:** Peter W. Halligan, David A. Oakley

**Affiliations:** ^1^School of Psychology, Cardiff University, Cardiff, United Kingdom; ^2^Division of Psychology and Language Sciences, University College London, London, United Kingdom

**Keywords:** consciousness, subjective awareness, cognitive neuroscience, epiphenomenon, non-conscious processing

## Abstract

Consciousness as used here, refers to the private, subjective experience of being aware of our perceptions, thoughts, feelings, actions, memories (psychological contents) including the intimate experience of a unified self with the capacity to generate and control actions and psychological contents. This compelling, intuitive consciousness-centric account has, and continues to shape folk and scientific accounts of psychology and human behavior. Over the last 30 years, research from the cognitive neurosciences has challenged this intuitive social construct account when providing a neurocognitive architecture for a human psychology. Growing evidence suggests that the executive functions typically attributed to the experience of consciousness are carried out competently, backstage and outside subjective awareness by a myriad of fast, efficient non-conscious brain systems. While it remains unclear how and where the experience of consciousness is generated in the brain, we suggested that the traditional intuitive explanation that consciousness is causally efficacious is wrong-headed when providing a cognitive neuroscientific account of human psychology. Notwithstanding the compelling 1st-person experience (inside view) that convinces us that subjective awareness is the mental curator of our actions and thoughts, we argue that the best framework for building a scientific account is to be consistent with the biophysical causal dependency of prior neural processes. From a 3rd person perspective, (outside view), we propose that subjective awareness lacking causal influence, is (no more) than our experience of being aware, our awareness of our psychological content, knowing that we are aware, and the belief that that such experiences are evidence of an agentive capacity shared by others. While the human mind can be described as comprising both conscious and nonconscious aspects, both ultimately depend on neural process in the brain. In arguing for the counter-intuitive epiphenomenal perspective, we suggest that a scientific approach considers all mental aspects of mind including consciousness in terms of their underlying, preceding (causal) biological changes, in the realization that most brain processes are not accompanied by any discernible change in subjective awareness.

“*Epiphenomenalism is counterintuitive, but the alternatives are more than counterintuitive*” (Chalmers, 1996, p. 160).

## Introduction

For something so obviously real and undeniable, ‘consciousness' was a late subject of formal psychological enquiry. This was due in large part to the compelling, common sense notion that as subjects we already know what ‘consciousness' is, by simply experiencing it. As such the function of consciousness is typically taken as self-evident in folk, philosophical and much of the psychological and neuroscience literature. Consequenctly for many scholars who drew the essential difference between first-person (subjective experience) and third person (scientific) accounts, the apparent challenge is to achieve an integrated explanatory framework that linked the intuitive subjective conscious experience with the rest of the natural sciences.

Another challenge for all researchers interested in providing an explanation for consciousness lies in is the realization that as a “mongrel concept” (Block, 1995) and “*a word worn smooth by a million tongues*” (Miller, 1962) there are many theories of consciousness, but none that are widely accepted.

While “*the term means many different things to many different people', [with] no universally agreed “core meaning*” (Velmans, 2009)… “*everyday experience furnishes the strong impression that conscious thoughts such as decisions, plans, and intentions play an important role in causing behavior*” (Baumeister et al., 2018).

Velmans suggest that some of the “*wide-ranging disparities* [in theories of consciousness] *arise more from pre-existing theoretical commitments (beliefs about the nature of consciousness, mind and world) than from the everyday phenomenology of ‘consciousness itself,”* although he assumes that the latter informs and shapes the former:

“*From a first-person perspective, consciousness appears to exert a central influence on human affairs, and scientists have a first-person perspective as much as their subjects. It is not surprising, therefore, that consciousness has been thought to enter into every major phase of information processing, ranging from the analysis, selection and storage of input to the organization, planning, and execution of response….[as such] …viewed from a first-person perspective, consciousness is central to the determination of human action.”* (p32/34, Velmans, 1991).

To help resolve some of the confusion arising from this “ill-defined concept,” the philosopher Block (1995) suggested a conceptual distinction between two interacting types of consciousness: *phenomenal consciousness* (P-consciousness) and *access consciousness* (A-consciousness). Phenomenal consciousness is the first person, immediate and non-reflective, experience of sensations, which describes feelings, perceptions, thoughts, wants and emotions, inaccessible to an external observer – and not requiring further justification.

Access consciousness, however, is characterized by reportable, representational content and involves the ability to self-monitor and engage cognitive functions such as reasoning and movements for direct control of action and speech.

Building on these views, traditional notions of “consciousness” comprise at least 3 related features

- the experience of subjective awareness (a component of P-consciousness)
- awareness of self together with the perceived volitional capacity to make decisions and control actions (a component of P-consciousness).
- the awareness of psychological states such as thoughts, attention, perception, intentions, memories, emotions - (A-consciousness -sometimes described as the “contents of consciousness”).

With regard to the A-consciousness, Baars and McGovern (1996; p. 91–92) suggest that “consciousness” covers a range of cognitive functions including:

- *Defining a stimulus and removing ambiguities in perception and understanding*.
- *Adaptative and Learning functions*.
- *Prioritizing and Access control functions when seeking high level goals*
- *Recruitment and Control of mental and physical actions*.
- *Decision-making and Executive functions*.
- *Error-detection and Editing functions*.
- *Reflective and Self-monitoring functions*.
- *Optimizing the trade-off between organization and flexibility*.

The intuitive, first person experience affords an easy and unquestioned attribution for many of the core cognitive functions including agency, body ownership and social responsibility. This promotes the wider concept of a personalized “free will” and unified self which in turn provides for the socially prevalent acceptance of libertarian, religious and democratic views. According to Pierson and Triut (2017) the ultimate adaptive function of consciousness is to make volitional movement possible, and all conscious processes exist to serve this ultimate function.

In the late 19th century, the reliance on introspection provided the early pioneers of psychology with the first systematic examination of conscious experience and an intuitive explanation. Citing the lack of scientific reliability, behaviorists later discarded this line of enquiry for nearly half a century. Recovered by cognitive psychologists in the 1960s using computational analogies, the main focus of the new-wave psychology, lay however, less in elucidating consciousness *per se*, than in charting the hidden information processing structures located in modular systems with “most psychologists” recognizing “*that they do not know what consciousness is*” [Miller, 1962 p. 27.]

Even by the 1970s and 1980s, when cognitive science become more established and confident, consciousness remained a much-neglected topic (Sohn, 2019), because the “dominant assumption of cognitive psychology in the 1970s was that the higher mental processes were almost entirely under conscious executive control” (Bargh, 2019) with some authors speculating that it was necessary for high level processes such as choice, learning and memory, and the organization of complex, novel responses, particularly those requiring planning, reflection, or creativity (Velmans, 1991). According to Kotchoubey (2018), most contemporary theories of consciousness consider consciousness to be a kind of executive information *processing* (e.g., Johnson-Laird, 1983; Dennett, 1991; Damasio, 1999; Edelman and Tononi, 2000; Koch, 2004; Maia and Cleeremans, 2005) without spelling out the detailed cognitive architecture of the executive system.

One explicit account that claimed an executive role for consciousness is Schacter's Dissociable Interactions and Conscious Experience (DICE) model (1990). This account proposed that “*the processes that mediate conscious identification and recognition – that is, phenomenal awareness in different domains — should be sharply distinguished from modular systems that operate on linguistic, perceptual, and other kinds of information*” (p. 160–161, 1990).

**Figure 1:**
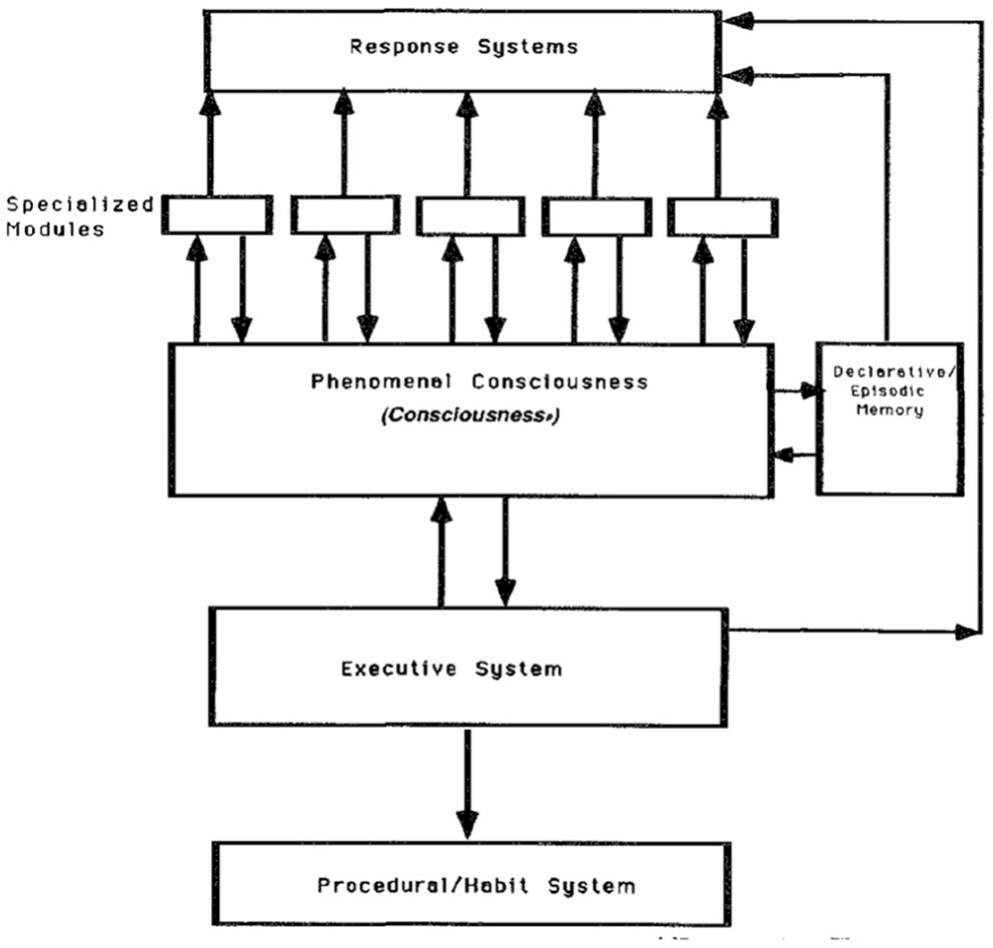
Schacter's Dissociable Interactions and Conscious Experience (DICE) model form (Block, 1995).

Similar to Shallice's Supervisory System (Shallice, 1988) account, Schachter's DICE model suggests that the primary role of consciousness as shown in Figure 1 is to mediate voluntary action under the control of a central executive. In this model, Schacter (1990) provides consciousness with a key information processing role responsible for integrating the outputs of specialized modules and transmitting the resulting signals to the executive system.

The belief that consciousness comprises a number of high-level cognitive, agentive controlled functions is found implicitly in most contemporary cognitive psychology and neuroscience accounts together with a qualitative split between conscious (intentional/within subjective awareness) and non-conscious processes (unintended/and outside awareness) (Dennett, 1991; Damasio, 1999; Edelman and Tononi, 2000; Koch, 2004; Maia and Cleeremans, 2005). This widely accepted dichotomy formalized within psychological theories has been described in terms of controlled vs. automatic cognitive processes (Norman and Shallice, 1986), slow vs. fast thinking (Kahneman, 2011), with the second process being the province of the “cognitive unconscious” (Kihlstrom, 1987).

The widespread acceptance of the binary difference helped motivate the growth in psychological and neuropsychology studies interested in discovering the underlying “impaired” (hidden) “non-conscious” psychological architecture. In this and previous articles, we draw no distinction between the terms unconscious and non-conscious.

## Challenging the Primacy of the Conscious-Centric Account

Bargh notes that “*until quite recently in the history of science and philosophy, mental life was considered entirely or mainly conscious in nature (e.g., Descartes' cogito and John Locke's “mind first” cosmology). The primacy of conscious thought for how people historically have thought about the mind is illustrated today in the words we use to describe other kinds of processes—all are modifications or qualifications of the word conscious (i.e., unconscious, preconscious, subconscious, non-conscious”* (Bargh and Morsella, 2008 p. 73).

In Halligan and Oakley (2000), we argued that this traditional conscious-centric account, although experienced as real, is from a science perspective wrong headed and that a more detailed consideration everyday phenomenological experience and evidence from cognitive neuroscience suggested that however compelling first person experience, consciousness has no explicit causal function and that this functional attribution relies on a powerful intuitive false belief, albeit one we are all strongly adapted to maintain. Why is this?

According to Graziano et al. (2019), our brain systematically misleads us into thinking that consciousness has non-physical, experiential, properties by constructing an inaccurate form of self-description, based on the brain's internal models for monitoring attention (Graziano, 2013; Webb and Graziano, 2015).

In 2017 (Oakley and Halligan, 2017), we presented a detailed account arguing that non-conscious neural causation provided a more plausible (albeit non-intuitive) basis to explain both the “experience of consciousness” and the “contents of consciousness.” We consider that subjective awareness provided no functional or causal impact upon psychological outputs or actions other than appearing contemporaneously with those psychological events as experienced.

Like others (e.g., Searle, 2000), we consider “consciousness,” to be a first-person experience generated in a similar way to other physiological process like respiration, circulation, and immune functions and where the contents of subjective awareness (thoughts, memories, perception, and action) are the result of selective neuro-cognitive processes, just as “digestion” is a product of selective and co-ordinated gastrointestinal processes.

Central to the different accounts of consciousness are attempts to reconcile the first person (private) and third person perspectives. However, these perspectives do not appear to comfortably inhabit the same *explanatory domains*. Implicit acceptance of the first person, private conscious-centric perspective continues to dominate attempts to develope a 3rd person scientific account with the resulting adherence to a compelling belief in agentive consciousness.

Although not surprising, everyday introspection suggests that the subjective nature of first-person awareness is not that of a qualitative experience constructed or controlled by us, but rather of a qualitative experience that is provided effortlessly, and which we have limited or little perceived control.

Consider how effortlessly we regain consciousness each morning after losing it the night before; how our thoughts, ideas and emotions arrive already preformed; how colors and shapes are constructed into meaningful objects or memorable faces without any deliberate effort until we become aware of them as meaningful objects.

As Velmans (2002) points out: “*One is not conscious of one's own brain/body processing. So how could there be conscious control of such processing? How “conscious” is conscious, voluntary control? … One might be aware of the fact that relaxing imagery can lower heart rate, but one has no awareness of how it does so, nor, in biofeedback, does one have any awareness of how consciousness might control the firing of a single motor neurone. One isn't even conscious of how to control the articulatory system in everyday “conscious speech!” Speech production is one of the most complex tasks humans are able to perform. Yet, one has no awareness whatsoever of the motor commands issued from the central nervous system that travel down efferent fibers to innervate the muscles, nor of the complex motor programming that enables muscular co-ordination and control. In speech, for example, the tongue may make as many as 12 adjustments of shape per second - adjustments which need to be precisely coordinated with other rapid, dynamic changes within the articulatory system. According to Lenneberg (**1967**), within 1 min of discourse as many as 10 to 15 thousand neuromuscular events occur. Yet only the results of this activity (the overt speech) normally enters consciousness”* (p. 6).

“*We usually remain unaware of many of our own actions. One reason is that, even when an action is consciously executed, its memory trace is of a very short duration, and so rapidly forgotten. It is indeed a common experience when leaving home to ask oneself “Did I lock the door?” or “Did I turn off the light?” immediately after doing it. One may also drive home and suddenly realize that one has arrived there without having the least idea how one did it.”* (Jeannerod, 2006, p. 25).

As all cognitive functions attributed to consciousness must derive from underlying neural “machinery” that give raise to them in the brain, we argue that to avoid the endless vacillation between 1st and 3rd person perspectives, when explaining human psychology from a scientific perspective, we suggest that subjective awareness (consciousness) possesses no cognitive function (i.e., has no functional utility) separate from the brain, any more than there is a specific category of “digestive” process that exists separate from the physicality and workings of the gut.

Despite this, most of us feel compelled to believe that when we make a voluntary action, we do not feel as though it has simply happened, but rather instead “feel” as though we are responsible for the action (Moore, 2016).

As such, our account can be seen to support Ryle's (1949) critique of the concept of a “*ghost in the machine”* as some form of qualitative, distinctive intervening mysterious state separate from the neural machinery and comprising cognitive representations providing for agentive and control processes. Thoughts and behavior are *caused* by brain activity – but being *by-products of the neural activity have no causal efficacy* for our behavior or on the activity of the brain itself.

Rather than being conceived as a mysterious immaterial “ghost” in the neural machinery, the reality, we suggest, is that we have subjective experiences (consciousness) generated by brain systems associated with feelings of agency and control. Having such feelings alone does not prove that subjective awareness directs or controls our psychological processes and outputs.

We see the brain as the ultimate and proximal computational information processing apparatus, that effortlessly generates our psychological contents and creates self-representations (including the familiar sense of “self”), our body image, as well as providing an ongoing personal narrative.

Accordingly, while accepting that one can draw a qualitative distinction between P and A consciousness, we hold that neither engage any cognitive executive functions *per se*. As such, the traditional parsing of psychological states in terms of the presence or absence of consciousness is a relative distinction as both are carried out by underlying brain systems. Perpetuating this distinction serves to constrain the scientific understanding of psychological processes by excluding the reality that non-conscious brain processes are responsible for *all* psychological processes including conscious awareness itself.

Given the indisputable physiological and pathological evidence for a causal dependency between subjective awareness and brain functioning, a central quest of modern neuroscience has been to identity & locate the *neural correlates* of subjective awareness (Crick and Koch, 1990; Koch et al., 2016). Underlying this search are at least 3 assumptions that:

- *subjective awareness is not generated by some mysterious immaterial substance*
- *subjective awareness is causally generated and dependent on neural processes*
- *subjective awareness does not causally generate or control psychological processes or outputs*

Accepting these assumptions, questions the belief implicit in most folk and scientific accounts of consciousness that links subjective awareness with proximal agentive causal functions.

In this paper, we present a brief description of an alternative model of cognitive processing which does not rely on consciousness as an intervening cognitive process. We then outline some consequences where the utility of the non-conscious neurocognitive processes take primacy when providing a scientific account where most neurocognitive changes are not reflected in subjective awareness.

We also consider the expanding role of the “cognitive unconscious,” and, related to that, the justification for an epiphenomenalist position regarding the nature of subjective awareness – which in effect involves removing the ghost from the operations of the neuro-cognitive machinery.

## The Oakley-Halligan Model

While accepting the reality of a subjective awareness, the Oakley and Halligan model (2017) is not intended to explain how or where neural processes produce consciousness, but rather to challange the common attribution of cognitive functions to consciousness and to offer an alternative framework in keeping with findings from cognitive neuroscience and introspection.

Based on experience from a range of psychological (Oakley, 1985) and neuropsychological phenomena including striking dissociations between what patients report verbally and what they can respond to behaviourally (e.g., visual neglect, anosognosia) (LeDoux et al., 2020), hypnosis (Oakley and Halligan, 2009, 2013), suggestion (Halligan and Oakley, 2014), placebo (Kirsh, 2019), and conditions such as amnesia (Milner, 1967), blindsight, (Weiskrantz, 1997), visual neglect (Marshall and Halligan, 1988; Halligan and Marshall, 1991), hysteria (Halligan, 2011), and phantom limbs (Halligan, 2002), we concluded that psychological content experienced in subjective awareness is generated by and within non-conscious brain systems in the form of a continuous self-referential “personal narrative.”

Wegner (2002) presents a compelling series of cases where people feel they are willing an act when they are not, such as in phantom limbs and ear wiggling, or when they feel they are not willing an act or experience when they are in fact initiating, such as auditory hallucinations, automatic writing, the Chevreul pendulum effect, dowsing, ideomotor actions – particularily in the context of hypnosis, which can produce the feeling that “*your actions are happening to you rather than that you are doing them*” (Lynn et al., 1990).

We propose that this continuously updated narrative arises from the “internal broadcasting” of selective outputs of cognitive processing, sensory information, and motor control. The personal narrative that “we” as individuals are familiar with forms the basis for autobiographical memories, but critically also serves the powerful evolutionary function that enables us to communicate the content of our internal broadcasting, all of which allows recipients to generate potentially mutually adaptive strategies.

We argue that it is the capacity to communicate the contents of these personal narrative either as knowledge or beliefs to other minds, and not personal awareness *per se* that confers individual evolutionary advantage. This transmission and sharing of internal narratives (external broadcasting) via language and other means has the potential to create a cultural reservoir of ideas and information accessible to the broader group as a shared resource. Importantly, both personal awareness and the personal narrative themselves are seen as passive non-agentive consequences of neurocognitive processing. Externally broadcast contents of the personal narrative, however, can serve an agentive function in initiating change in others in a social/cultural context.

**Figure 2:**
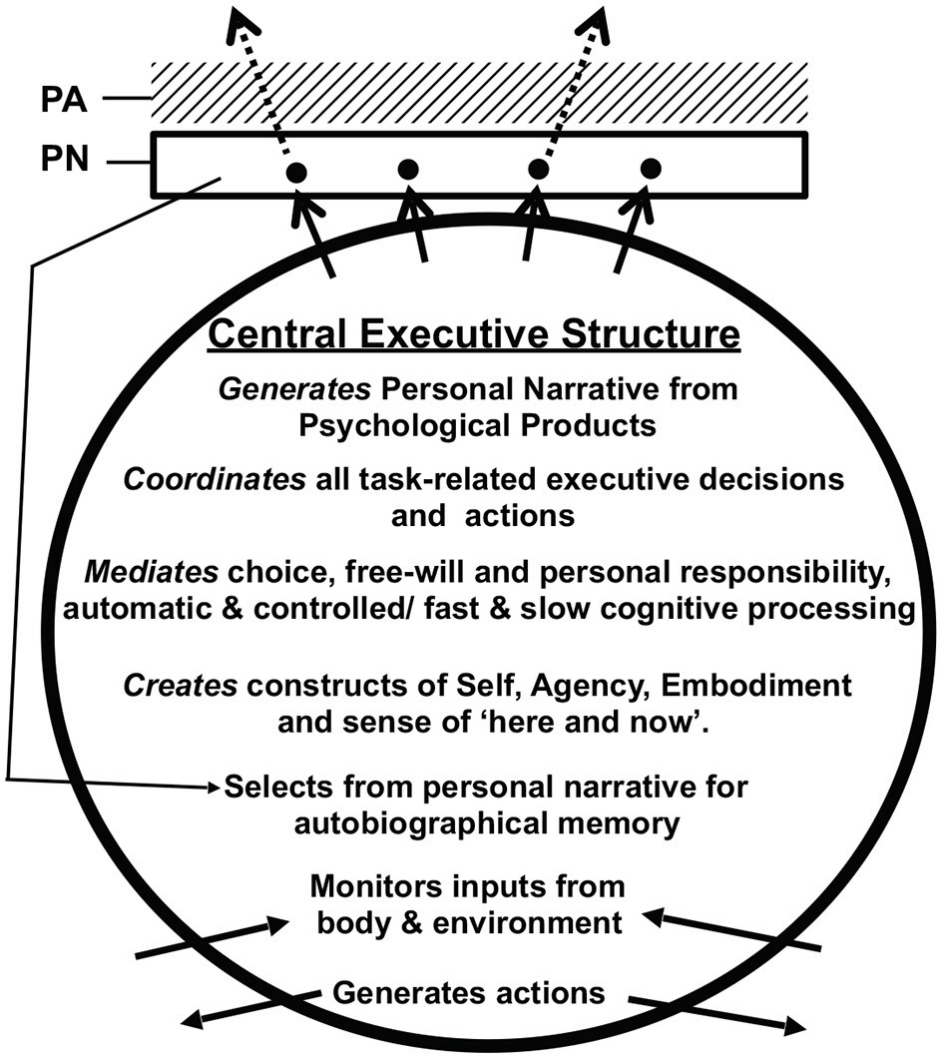
Abbreviated version of the Oakley-Halligan model of cognitive psychological function. For the full version see Oakley and Halligan (2017). The Central Executive structure creates and orchestrates all the cognitive psychological processes carried out by the brain. The solid arrows at the top of the model represent the process of Internal Broadcasting that underlies the creation of an ongoing Personal Narrative (PN). The dotted arrows represent the process of External Broadcasting whereby selected contents of the personal narrative can be communicated particularly, but not solely, via language, to other individuals facilitating social collaboration and the creation of a cultural store of ideas, skills, and information. Personal Awareness (PA) is an epiphenomenal subjective experience that passively accompanies the contents of the personal narrative. Also shown is the linkage between contents of the PN and autobiographical memory.

This conceptual reframing of subjective awareness as a passive accompaniment to neurocognitive processes, offers a reset and focus for elucidating the cognitive architecture when exploring the origin and function of psychological processes, previously attributed in large or small part to the presence of an elusive executive “consciousness.” A simple schematic model of proposed cognitive psychological processing in the brain is shown in Figure 2 (See Oakley and Halligan, 2017, for a fuller account).

In brief, the core Central Executive Structure (CES) is a functional collection of neurological systems used to coordinate and select psychological products from different brain systems mediating attention, perception, memory, reasoning, Language, problem solving, planning, theory of mind, and the search for meaning. The psychological products of these neural systems include thoughts, beliefs, ideas, percept's, sensations, speech, memories, feelings, intentions. actions, reflexes, and habits.

The operative assumption here is that psychological states taxonomized by appeal to their psychological properties are identical with neural states and processes, and that these non-conscious states (being outputs of underlying neural processes) have utility in shaping the psychological contents and actions that make it into subjective awareness.

The CES is responsible for creating the continuously updated personal narrative (PN) from components of brain activity considered most relevant for an ongoing task. The process of creating the personal narrative we describe as “internal broadcasting.” The personal narrative is accompanied passively by subjective experience – personal awareness (PA).

Within this model PA, unlike the non-conscious neuropsychological processes, is epiphenomenal and its neural origins remain to be determined by further scientific research into comparable neuro-cognitive processing systems. Accordingly, PA is not able to influence the brain process associated with it. An important feature of CES activity it that it also able to select some contents from the personal narrative for transmission to other individuals primarily by speech in humans but also via writing, gestures and other non-verbal media. This second transmission process we refer to as “external broadcasting.” This facilitates social collaboration and the creation of a cultural store of ideas, skills and information - see Oakley and Halligan (2017) for further details.

In common with Graziano's account (2017) we envisage that the brain generates a range of higher-level representational schemata, identified in our case as the CES, capable of exerting control over lower level schemas as well as more basic level functions, but not dependent upon personal awareness. We don't assume an over-arching schema that controls other schemata – though we assume this is the way the brain is represented via internal broadcasting of cognitive processes into the PN. Even if this was the case, then paradoxically, in traditional terminology it would have to be categorized as non-conscious consciousness.

An understandable objection to the phenomenological account is that as human beings we have developed extensive social and legal governance structures that explicitly assume a conscious-centric account that actively promotes and accepts the case for individual responsibility, mental causation and self-directed actions. To substantiate the belief in “free will” (the power or capacity of a “self” to choose among alternatives and to act in situations independently of biological or social restraints) one could argue that one needs to hold with the belief that humans have conscious centric mediated systems designed for self-directed “voluntary” abilities. In other words, the concept of consciousness is needed to express the delusion of free will (Meese, 2018).

An epiphenomenalist could argue (Bacrac, 2010) however, that the subjective experience or feeling of exercising “choice” is not dissimilar to that of the subjective experience of thinking or remembering and other psychological outputs. As psychological outputs derived from underlying cognitive systems are ultimately dependent on brain processes, first person awareness is not privy to how these feelings or psychological outputs are generated, and only become aware of them when they are expressed or “come to mind” with the feeling that we choose.

Bignetti (2014) provides a helful analysis of this persistent and deep-rooted subjective belief as it relates to free will. A “voluntary” action is performed by a neurocognitive system which always precedes the experienced activity. Unaware of the temporal lag and non-conscious processing that precedes such subjective awareness; we as subjects erroneously believe that we freely decide the action – which satisfies and confirms the psychological need for the sense of agency and personal responsibility.

While the subjective experience of free will ultimately depends on the selective involvement of underlying neural processes and their cognitive instantiation, it is often assumed that subjective awareness has the functional capacity to exercise free will despite that fact that research has shown that people can interpret randomness in their own and others' behavior as owing to free choice (Ebert and Wegner, 2011).

Recognizing that most legal and political systems take the causal efficacy of conscious mental states for granted, we argue that belief in free will and personal responsibility evolved as protective social constructs (Halligan and Oakley, 2015) not dissimilar from the way our brain creates subjective awareness of body ownership, sustains our key body processes (such as respiration, blood circulation, digestion etc.) and also generates outputs from our dedicated psychological processes (attention, memories, colors, shapes, calculations, speech).

In our account (Oakley and Halligan, 2017) the social constructs of free-will, choice, and personal accountability, are not dispensed with—but are assumed to be embedded in non-conscious brain systems where their existence as near universal constructs serving powerful social purposes are maintained through cultural broadcasting impacting via individual nervous systems on personal narratives.

## The Natural Primacy of Non-conscious Processes

Our non-conscious centric account of consciousness critically builds on the work of Bargh and Chartrand (1999), Bargh and Williams (2006) and Bargh (2008) who have long argued that much of contemporary psychology's intuitive assumption regarding the primacy of consciousness is wrong-headed and that the study of psychology needs to keep in mind, that in nature, the “unconscious mind” tends to be the rule and not the exception.

Bargh (2008) argues that despite the fact that “*the history of social psychology, and especially its subfield of social cognition, is replete with surprising findings of complex judgmental and behavioral phenomena that operate outside of conscious awareness and even intention (Wegner and Bargh*, *1998**). … the surprising nature of these findings comes no longer from their relative infrequency,.but from the continuing overarching assumption of the field regarding the primacy of conscious will” (p. 128)*.

Bargh (2008) suggests that this assumption arises from our own subjective experience whereby “*the early process models of each new phenomenon tend to start with the assumption of a major role played by conscious choice and decisions, intention and awareness, in producing the phenomenon in question*” (p. 128).

Bargh (2008) also reminds us that “*before there was consciousness, there already were all the unconscious modules and components that evolved to serve adaptive ends—selective sensitivity to the important and dangerous aspects of the environment… basic motivations to survive, eat, reproduce, to avoid what was known to be bad for us and to approach that which was good for us*” (p. 144)

This perspective is also captured by the philosopher Jackson (1996). “* …we can explain the way plants non-accidentally orient themselves toward the sun in terms of how their internal states get appropriately modified by the direction of the sun's rays on the plant before the corresponding movement. When we open up the plant, we find the state that does the work, and also find how it is sensitive to the sun's direction in such a way that the plant orients itself appropriately toward the sun. It would be a mistake for philosophers to write to biologists telling them that the internal states that they cite in their texts cannot, as a matter of principle, explain the relational nature of the movements of plants. The same goes for philosophers writing to animal biologists – and we are animals” (p. 391–392)*.

Similarly, Dennett (1991, p. 251) noted, “*in biology, we have learned to resist the temptation to explain design in organisms by positing a single great Intelligence that does all the work. We must build up the same resistance to the temptation to explain action as arising from the imperatives of an internal action-orderer who does too much of the specification work*.”

Since non-conscious brain systems carry out a myriad of complex core biological processes effortlessly and efficiently, non-conscious causation provides a more plausible (albeit non-intuitive) account to explain the traditionally attributed “*contents of consciousness*” and the concurrent “*experience of consciousness*.”

Moreover, such an approach is consistent with the observation that, “*in the rest of the natural sciences, especially neurobiology, the assumption of conscious primacy is not nearly as prevalent as in psychology. Complex and intelligent design in living things is not assumed to be driven by conscious processes on the part of the plant or animal, but instead by blindly adaptive processes that accrued through natural selection”* (Bargh and Morsella, 2008, p. 78).

Science is no stranger to conceptual misperceptions and new paradigms of explanation replacing older accounts. Consider “spontaneous generation” (the belief that life regularly arose from the elements without first being formed through a seed, egg, or other traditional means of reproduction) or the previous well-accepted geo-centric model of the universe (from Ptolemy), subsequently replaced by Copernicus's sun-centered astronomical model (the heliocentric account) effectively removing Earth from the center of the then known universe. Neuroscience examples include the nature of neglect, phantom limbs and the experience of pain (Melzack and Wall, 1965).

The development of cognitive accounts of visual neglect was delayed by the longstanding notion that the condition was due and could be explained as resulting from primary sensory disorders. Before visual neglect became more commonly appreciated in behavioral neurology and cognitive neuropsychology as an attentional/representational disorder, the condition was often interpreted as a subset of clinical behaviors not explicitly distinguished from those attributed to unilateral sensory disturbances (Bender, 1952; Battersby et al., 1956; Eidelberg and Schwartz, 1971; Weinstein and Friedman, 1977).

As Cohen (1993, p. 194) noted:“*Many neurologists prior to 1950 did not distinguish disorders of neglect from other unilateral sensory disturbances. The neglect of one side of the body or environment that some patients with contralateral brain lesions exhibited was considered to result from deafferentation of sensory pathways and or sensory cortex. This position was supported by the high incidence of primary sensory disturbances in these patients…therefore the unilateral quality of neglect seems to fit with the unilateral nature of the many sensory disturbances, in which the ascending sensory pathways are disturbed unilaterally.”*

It was only after a growing number of patients with lateralized neurological symptoms failed to show evidence of “primary deficits” using conventional neuro-optometry and neurological methods that neuropsychologists in the 1970s began to more confidently assert that VSN was a “higher-level disorder” that could not be attributed to the effects of “primary deficits” (Halligan and Marshall, 2002).

The experience of phantom limb following amputation in another example. This was long considered as counter-intuitive and anomalous (see Melzack, 1992; Halligan, 2002), which had been explained for some time as the product of a miraculous form of limb restoration before the 19th Century, thereby avoiding the necessity to challenge the compelling folk account that it was not possible to feel a body part that was no longer physically present. A source of this misconception according to Melzack (Melzack, 1992; Saadah and Melzack, 1994) was the assumed primacy of sensory feedback and a passive brain, arguing instead that “*Sensory inputs merely modulate that experience; they do not directly cause it*” and that “*Phantoms become comprehensible once we recognize that the brain generates the experience of the body.”*

In the field of visual perception, the investigation of “source-monitoring” by Kunzendorf (2020) provides a good example of the illusory nature of perceptual processes commonly attributed to consciousness. In these studies, participants viewed a picture or printed word on one side of their fixation point while visually imagining the same word or picture on the other side. They then rated the vividness of their self-generated image compared to the physical image and were requested to indicate which of the two images was their own creation. Surprisingly participants' ability to identify the imagined stimulus increased as their subjective rating of the vividness of the imagined image approached that of the actual stimulus. This indicated that their response was not based on a subjective perceptual difference between the two images, but as suggested by Kunzendorf (2020) on an internal, non-conscious, process of monitoring the biological source of the two images. Perception of the “real,” physical object being based on peripheral information from appropriate sensory systems, whereas imagined images relied on memorial brain activity stemming primarily from higher cortical centers. The experimental result is consistent with the view that “non-conscious” brain processes actively monitor “sources” of the information processing activity underlying the perceived image and rely on evidence from increased corticofugal activity to identify and hence distinguish the imagined, self-created image. Interestingly, the reverse pattern was seen with hypnotic suggestion, as well as in cases of the psychosis and dissociation-prone participants – whereby the individual's capacity for source-monitoring was explained as impaired and hence giving rise to a reduced ability to distinguish real from self-generated images.

To better align psychology as a science with the rest of the natural sciences, we should “*begin with the assumption of mainly unconscious instead of conscious causation of action and phenomenal experience”* (Bargh, 2008; p. 148).

## Relocating the Central Role Played by Non-conscious Brain Systems

According to Bargh and Morsella (2008), the long standing intuitive conscious-centric account began to be challenged in the 1800s, following “*two very different developments—hypnotism and evolutionary theory”*—both of which indicated the “*possibility of unconscious, unintended causes for human behavior*.”

Kihlstrom (1995) suggested that the German philosopher Eduard von Hartmann's published work, *Philosophy of the Unconscious* in 1869, was largely responsible for popularizing the concept of the unconscious, and that the notion of unconscious was in many ways superior to consciousness.

According to Hartmann “*… if in man we consider the sphere belonging both to the unconscious and also to consciousness, this much is certain, that everything which any consciousness has the power to accomplish can be executed equally well by the Unconscious, and that too always far more strikingly, and therewith far more quickly and more conveniently for the individual, since the conscious performance must be striven for, whereas the Unconscious comes of itself and without effort” (Hartmann*, *1868/1931* *Vol. 2, p. 39)*

Although initially criticized by William James and Hermann Ebbinghaus, Von Hartman's *Philosophy of the Unconscious* was widely read and with translations from German into French and English helped to make the idea of the unconscious both familiar and more acceptable (Kihlstrom, 1995).

At the close of the 19th century, the French neurologist Jean-Martin Charcot proposed the idea that unconscious brain processes were responsible for the unexplained neurological symptoms of hysteria and the pseudo-neurological behaviors commonly produced by hypnosis (Oakley, 1999b). Central to Charcot's explanation was the concept that symptoms could derive from unconscious “fixed” ideas based on suggestions or autosuggestions “*remaining isolated from the rest of the mind and expressing themselves outwardly through corresponding motor phenomena*” (quoted from Ellenberger, 1971 in Halligan, 2011).

Also, toward the end of the 19th century, the early pioneers of experimental psychology, Wundt, Titchener and Helmholtz, similarly recognized that areas of mental life, for which consciousness was claimed, were in fact the product of prior levels of non-conscious processing. Helmholtz (1924) regarded “unconscious processes” as “*the stuff of which conscious experience is made*” and where “*conscious perceptions are determined by unconscious inferences mental computations of which we can never be aware, and over which we have no control*” (Kihlstrom, 1995).

This idea was elaborated further and popularized in Sigmund Freud's psychodynamic theories in the 1920s, highlighting the potential and neglected powerful role that unconscious processes play in shaping human awareness and controlling everyday behavior (Oakley, 2012).

Unfortunately, however, “*just when the concept of the psychological unconscious was getting up steam, the behaviorist revolution hit-and the psychological unconscious went the way of consciousness itself. It was bad enough to explain behavior in terms of mental states that could not be publicly observed; and so, it was doubly bad to explain behavior in terms of mental states that could not even be privately observed!”* (Kihlstrom, 1995).

## The Cognitive Unconscious

As a formal area of cognitive enquiry, the study of consciousness remained largely neglected throughout most of the 20th century (Crick and Koch, 1990), despite renewed interest in the 1960s by a number of pioneers of the cognitive psychology revolution detailing informational-processing accounts of the key psychological processes of memory, perception and language comprehension.

The position was well captured by George Miller, one of the founders of cognitive psychology, who in 1962 pointed out that when one recalls something from memory “*consciousness gives no clue as to where the answer comes from; the processes that produce it are unconscious. It is the result of thinking, not the process of thinking, that appears spontaneously in consciousness*.” Chomsky (1980) later argued that language processing was largely performed by a set of structures and processes whose operation was completely inaccessible to consciousness (See Kihlstrom, 1995) and even later by Fodor (1983) who extended this notion by invoking cognitively impenetrable modules for designated domains such as visual perception.

However, it was Kihlstrom's seminal 1987 paper (and later work, Kihlstrom, 1996) that really highlighted the role and significance of unconscious, automatic psychological processes in perception, memory, and action and making it clear that “*conscious awareness. is not necessary for complex psychological functioning*” (p. 1450).

Around the same time, confirmatory research from social psychology received attention in the form of the much-cited paper by Nisbett and Wilson (1977) “*Telling more than we can know: Verbal reports on mental processes*.” This argued that we are only consciously aware of the products of our mental processes and have little or no conscious awareness of the processes themselves.

The eventual realization that so many aspects of mental life represent products of prior levels of “unconscious” processing continues to provide one of the reasons for the existence of a cognitive psychology in the first place. As Velmans (2000) points out:

“*if the complex processes which enable us to select information, attend to it, plan, organize, determine priorities, respond appropriately and so on, were available to consciousness, there would be no need for careful experiment and theoretical inference to determine their operations” (p. 68)*.

Despite growing evidence for an increasingly large, sophisticated and competent non-conscious system, for many researchers, consciousness remained relevant for explaining many agency aspects of mental life, and it was not regarded as “*an epiphenomenon …or passive correlate of brain processes, but rather an active integral part of the cerebral process itself, exerting potent causal effects in the interplay of cerebral operations*” (Sperry, 1977).

However, for others striving to accommodate the growing challenges from cognitive neuropsychology, introspection and neuroscience, a more nuanced, position began to be adopted. This ranged from a position where “*most human behaviors, especially most meaningful actions are the result of both conscious and unconscious processes, both of which are neurologically represented in the brain*” (Baumeister et al., 2011, 2018) to those like Bargh and Williams (2006) and Hassin (2013) who argued that unconscious processes can carry out almost every fundamental high-level function that conscious processes can perform and that the role of conscious is “*if not utterly zero, at least quite miminal and peripheral*” (Baumeister et al., 2018).

The 1960s witnessed renewed emphasis on informational processing accounts for memory, perception and language comprehension, however the cognitive enquiry into consciousness remained largely neglected (Marcel and Bisiach, 1988). It was “*the norm for accounts of personal experience to be used only informally*” (Shallice, 1988) and “.*most theories of cognition make no call at all upon consciousness*” (Marcel, 1988). Neither Shallice's (1988) nor Johnson-Laird's (1983) formal cognitive models implied “*a definitive causal role for consciousness*” (Bisiach, 1988).

Higher-order theories of consciousness such as Rosenthal (2008), suggest that mental states are only conscious, when and if one is aware of being in such a mental state. These higher order thoughts were distinguished by being reportable and recognized as belonging to us. Rosenthal avoided *epiphenomenalism* however by arguing that while consciousness of such intentional states lack significant function, this did not “*imply that the consciousness of these states has no causal impact on other psychological processes” but rather that the “impact is simply too varied and insignificant to yield stable beneficial effects”* (p. 831).

Studies from Libet et al. (1982) and later others (Soon et al., 2008; Rigoni et al., 2013) suggested that the intention to act always occurred later than preparatory brain activity (readiness potentials) in motor systems of the brain. This was taken as demonstrating that awareness of the decision to move and preparation of that movement was produced by previous non-conscious processes. As Gray (2004) concluded the experience experience of conscious intention comes too late to be the proximal initiator of the motor act, although sufficiently close in time to provide support for the intuitive causal account. Evidence from Haggard and Eimer (1999) showed that timing of the readiness potential and experience of the intention to move was non-linear, suggesting the two were largely independent.

By the 1990's, there was a growing number of neuroscientists and psychologists questioning the assumed role that consciousness played in our mental life (Velmans, 1991; Bargh and Chartrand, 1999; Hassin et al., 2005 for a detailed review see Earl, 2014). These included Dehaene and Naccache, 2001; Gray, 2004; Hassin et al., 2005; Eagleman, 2011; Hassin, 2013; Morsella et al., 2016).

## Challenging the Consciousness-Centric Position

Over the past 30 years, several converging lines of psychological and neuropsychological enquiry have questioned the assumed agency and functional role attributed to the experience of consciousness (or subjective awareness). Central to this challenge is the description of a number of dissociations demonstrating intact or residual cognitive abilities, despite subject's manifest lack of awareness, as in the case of patients, with neglect, dyslexia, blindsight, implicit memories, prosopagnosia, hypnosis, (Oakley, 1999a) and conversion hysteria (Halligan and Marshall, 1997).

In 1974, Weiskrantz described the phenomenon of “blindsight” (Sanders et al., 1974) where patients' actions appear to be guided by sensory information that they were unaware of, challenging the belief that perceptions must enter “conscious awareness” to affect or produce our actions. Similarly in striking cases of visual neglect, patients showed impressive non-conscious processing for stimuli on the neglected side of their visual fields, including object identification despite lack of reported visual awareness (Marshall and Halligan, 1988; Driver and Mattingley, 1998).

So why the long standing and persisting belief that subjective awareness comprises a cognitive agentive *internal action-orderer* with the capacity to make decisions and control actions and the awareness of psychological states such as thoughts, attention, perception, intentions, memories, etc?

Graziano's attention schema theory (2017) provides a possible explanation for this intractable belief in the form of an attention data-handling method employed by the brain to ration processing resources. According to this theory, the experience of consciousness arises from the brain's own feedback when monitoring attention. In the same way that the brain constructs a “body schema” that models interactions from touch, vision, and proprioceptive information to build a virtual model of a phantom body (see Halligan, 2002), Graziano suggests that the brain employs an analogous “attention schema,” that provides our cognitive processes with a high level (albeit imperfect) overview of where attention is distributed and where consciousness is “phantom's attention” (Graziano, 2016b).

According to Graziano et al. (2019) “*the belief in a non-materialistic component to the mind—is a lingering fragment of a larger cluster of incorrect culturally widespread, folk-psychological beliefs….that derive from implicit, social-cognitive models, that have infiltrated the science of consciousness' perpetuating mistaken assumptions*” (p. 3).

Evidence from cognitive development also suggests that such intuitive pre-instructional beliefs feature in many areas of science and often coexist potentially delaying or obstructing the acceptance of scientific accounts (Shtulman and Valcarcel, 2012; Shtulman and Harrington, 2016).

According to Shtulman and Harrington (2016) this conflict where participants continue to hold on to intuitive theories persists across the lifespan and can influence scientific reasoning for decades beyond the acquisition of a mutually incompatible scientific theory.

“*For instance, students charged with learning a kinetic theory of heat must un-learn a substance-based theory in which heat is construed as an immaterial substance that flows in and out of objects and can be trapped or contained (Reiner et al.*, *2000**). Students charged with learning a selection-based theory of evolution must unlearna need-based theory in which evolution is construed as a process that guarantees organisms the traits they need in order to survive (Shtulman and Harrington*, *2016**). And students charged with learning an inertial theory of mechanics must un-learn an “impetus” -based theory in which objects are assumed to move only if imparted an internal force, or impetus, andwill remain in motion until that impetus dissipates (McCloskey et al.*, *1983**)” (p. 119)*

“*These intuitive accounts are actively reinforced by how we talk about natural phenomena in everyday discourse and how we perceive natural phenomena in everyday situations…Research has shown that adults exhibit cognitive conflict when retrieving scientific information that contradicts the intuitive theories they had presumably abandoned as children*” (p. 132).

*One explanation for why intuitive theories seem to persist across the lifespan is that they may be represented in the brain in a cognitively impenetrable format, similar to the seemingly impenetrable representations of language (*Coltheart, 1999) and vision (Pylyshyn, 1999*). In vision, for instance, we can be well aware that our eyes deceive us when viewing the Muller-Lyer illusion or the Ponzo illusion, but we perceive the illusion nonetheless* (p. 132).

Much of our colloquial language predicates these persisting intuitive conceptions:

*The terms “sunrise” and “sunset,” for instance, imply that day and night are caused by movements of the sun rather than movements of the earth. More accurate terms would be “sun accretion” and “sun occlusion.” Likewise, the terms “warm coat” and “cold wind” imply that heat is an intrinsic property of objects rather than something that is transferred across physical systems. More accurate terms would be “insulating coat” and “disequilibrating wind.”* (Shtulman and Harrington, 2016, p. 133)

Arguing that subjective awareness (consciousness) and mental phenomena are a direct consequence of neural process, clearly speaks against the commonly held intuitive dualist accounts where consciousness is perceived as a critical contributor when explaining human psychology.

Research from developmental psychology suggests that while the precise formulation of such beliefs are culturally determined, “*the idea that consciousness is different from the body is universal.”* Eagleman (2011).

Using survey evidence where the majority of respondents considered the mind was not purely a physical entity, Demertzi et al. (2009) suggest that belief in dualism is widespread and continues to exert an influence on scientific thought, whereas “*cognitive neuroscience reflects a sustained attempt by scientists to re-instate mind within nature, from which it was exiled by Descartes at the inception of modern science”*(p. 8).

According to Webb and Graziano (2015), the internal read out from the attentional schema is “*the brain's way of rationing its processing resources,”* that leads the brain to conclude that it has a subjective experience. Hence, according Graziano (2016a) “*the human brain insists it has consciousness, with all the phenomenological mystery, because it constructs information to that effect.”*

Unlike Graziano (2013), who considers consciousness as playing “*an active role in guiding our behavior*,” we consider subjective awareness to be epiphenomenal, in much the same way that the redness of blood is a consequence of the biological mechanism required to deliver oxygen, but not a function in its own right.

In adopting this epiphenomenalist explanation for the experience of awareness, we propose (Halligan and Oakley, 2015, 2018; Oakley and Halligan, 2017) that the functional capacities commonly attributed to it are generated non-consciously by cognitive processes from a neurally instantiated central executive system. As such, we consider subjective experience (“personal awareness” in our model) to be a passive, accompaniment to a non-agentive personal narrative, created by the selective “internal broadcasting” of task-related outputs from non-conscious neural executive systems that have access to cognitive processing, sensory information, and motor control.

Consequently as “subjects of unconscious authoring” (Meese, 2018), our account makes no reference to consciousness as an agentive controller – rather we propose that this subjective personal awareness is a passive accompaniment to the task-relevant cognitive events that form our “personal narrative” and exerts no causal or controlling relationship over any psychological processes or contents.

Our account questions the strong notion of a binary distinction between conscious and non-conscious mental systems and suggests the need to reframe the traditional qualitative distinction in favor of a continuum of non-conscious processes (Halligan and Oakley, 2018).

## Revisting Epiphenomenalism

The conceptual foundations of our account have existed since at least the 19th century and can be characterized as epiphenomenal property dualism, a counter-intuitive philosophical position that considers the world as comprising one physical substance (e.g., body/brains) but having two fundamentally different properties, mental and physical, and where mental phenomena are solely generated (caused) by the physical substance (i.e., where mental properties are “causally redundant”).

Accepting this property dualism, all mental states (our thoughts and feelings, and sense of self) are generated by electrochemical brain activity and remain dependent on such brain states. Without a brain or relevant brain states there are no mental states.

Although we readily accept the first-person reality of personal experience, we aver a uni-directional causal relationship, whereby neurological states (somehow) generate mental states, but that these mental states (derived from physical states) that we become aware of, do not have the capacity to influence physical or mental states. As Robinson (2010) points out “*the less we feel we can rely on our intuitions in this area, the more seriously the claims of epiphenomenalism have to be investigated.”*

The neurocognitive states that provide for mental states have a direct effect on both the experience (i.e., subjective awareness) and the contents of that awareness (feelings, thoughts, agency, self, etc.).

Neuroscience has yet to explain *how* subjective awareness is generated by brain systems. However, there is compelling evidence from everyday observation, neurology, neurophysiology and cognitive sciences that demonstrate that the existence and proper functioning of subjective awareness and associated psychological processes is causally dependent on the intactness and interplay of different brain regions.

The current explanatory gap (how neural systems generate mental states) does not of itself provide grounds in the belief that mental processes can direct psychological and corporeal systems. Whatever subjective awareness is – it has nonetheless to be fundamentally linked to physiological alterations in the central nervous system. All mental operations require the temporal precedence of physiological processes before outputs are experienced in conscious awareness. There is no evidence that mental states can exist or are capable of being experienced without functioning brain systems.

To help counter the apparent absurdity of the epiphenomenologist position when applied to the perceived exercise of conscious will, Wegner developed a theory of Apparent Mental Causation (Wegner and Wheatley, 1999) which argued that “*people experience conscious will when they interpret their own thought as the cause of their action.”* According to the theory, “*when a thought appears in consciousness just prior to an action, is consistent with the action, and appears exclusive of salient alternative causes of the action, we experience conscious will and ascribe authorship to ourselves for the action”* Wegner (2004, p. 1). In other words, the experience of conscious thoughts and actions are produced in parallel—and both are generated by unconscious neural events.

According to Wegner's theory, when we make voluntary actions, there is an *unconscious* causal motor pathway responsible for the action. This pathway corresponds to the workings of the motor control system. In addition, there is also an unconscious causal pathway responsible for the associated thoughts about the actions (i.e., a link back to our intentions). According to Wegner and Wheatley (1999) it is the relationship between the thought and the action that produces the *sense of agency* (or in Wegner's term, the “experience of conscious will”). When the intention to act happens before we act, and is consistent with the action, and is the only plausible cause of the action, then we feel as though we have caused the action.

In support of this view, Wegner cites evidence from “*clinical disorders such as alien hand syndrome, dissociative identity disorder, and schizophrenic auditory hallucinations”* and non-clinical case examples including phenomena from “*hypnosis, automatic writing, Ouija board spelling, water dowsing, facilitated communication, speaking in tongues, spirit possession, and trance channeling*” (Wegner, 2004, p. 1).

Wegner also cites the pioneering studies of Penfield - one of the first neuroscientists to experimentally map basic sensory and motor areas during brain surgery while patients were conscious and showed how selective brain stimulation could cause a person's hand to move without their experience of volition.

According to Wegner (2002) the evidence that brain stimulation could produce voluntary-appearing actions without the actions performed being felt as consciously willed.…* suggests that the brain structures that provides the experience of will is separate from the brain source of action. It appears possible to produce voluntary action through brain stimulation with or without an experience of conscious will. This, in turn, suggests the interesting possibility that conscious will is an add-on, an experience that has its own origins and consequences…only loosely coupled with the mechanisms that yield action itself (*Wegner, 2002: 47).

The strongly held intuitive belief in an agentive subjective awareness capable of generating and/or influencing psychological states is seen as originating from brain systems which are ultimately responsible for both generating the contents of our psychological states and the experience of subjective awareness.

Adopting the epiphenomenalist stance, the experience of consciousness (subjective awareness) is a product of unconscious brain processes. The compelling belief in its causal subjective abilities arises (not unreasonably) from the close association whereby mental events – are temporally associated –and are therefore perceived to produce the behaviors or actions that follow.

An early version of *epiphenomenalism* articulated by the biologist Huxley (1874) noted that “*All states of consciousness in us,[…], are immediately caused by molecular changes of the brain-substance. It seems to me that in men (sic), as in brutes there is no proof that any state of consciousness is [itself] the cause of change in the motion of the matter [brain] of the organism*.”

The philosopher David Chalmers (1996) points out, that as the *epiphenomenalist* position is strongly counter-intuitive as an explanation for everyday lived experience it is not surprising that it has few friends (Jackson, 1982; Baysan, 2020), and has been deemed “*a disgrace… more awful than dualism*” (Honderich, 2001, p. 247, 278); “*a dreaded relic* of pre-scientific philosophy” (Dennett, 1998) “*thoughtless and incoherent”* (Taylor, 1927, 198), “*unintelligible”* (Benecke, 1901, 26), “*quite impossible to believe”* (Taylor, 1963, 28) and “*truly incredible”* (McLaughlin, 1994, 284).

On the other hand, “*the vigor of the opposition to it, however, is a backhanded recognition that there are substantial considerations that at least appear to support it*” (Robinson, 2010).

Many findings of “s*cience are typically counter-intuitive and probabilistic*” (Dela Sala, 1999) and “*novelty emerges only with difficulty, manifested by resistance, against a background provided by expectation*” (Kuhn, 1970). The astronomer Nicolas Copernicus (1473–1543) published his book in 1543 challenging the established geocentric model of the universe, a position that lay at the heart of the Aristotelian and religious worldview and remained unchallenged for centuries. In 1616, the Catholic Church issued a prohibition against the Copernican theory of the earth's motion, and this resurfaced at the Inquisition trial and condemnation of Galileo Galilei in 1633.

According to Lane and Harris (2014) “…*children and adults believe in a variety of entities and processes that defy and overcome intuitive conceptions and first-hand perceptions of the world. These include scientific concepts of invisible germs and oxygen, heliocentrism, and a spherical Earth, as well as religious concepts of deities who are all-knowing and all-powerful, Heaven, and souls that continue to exist even after the mind and body have died.”*

Epiphenomenalism is not a natural, intuitive explanation and is incompatible with our own first-hand personal experience. However, Baggini (2005) in his fictious “Land of the Epiphens” provides a helpful story that captures both our “naturalistic” or default thinking (Hassin, 2013), while also highlighting the challenge of confusing correlation with causation.

Baggini's (2005) allegory (quoted below) illustrates the way in which inhabitants of Epiphenia and humans might interact on the subject with the key difference however being that “epiphens” believe that thoughts do not serve as causes:

“*Epiphenia was a remarkable planet. So like the Earth in appearance, and yet its inhabitants were different in one remarkable way.”*

*As one of them, Huxley, explained to the visiting Earthling Dirk, the Epiphens had long ago “discovered” that their thoughts did not affect their actions. Thoughts were the effects of bodily processes, not the other way around. Dirk found this baffling*.

‘*'You can't really believe this,” he protested to Huxley. “For instance, when we met in this bar, you said, “Gee, I could kill for a beer,” and ordered one. Are you saying that the thought “I want a beer,” had no effect on your actions?”*

“*Of course it didn't,” replied Huxley, as though the question were idiotic. “We have thoughts and these often precede actions. But we know full well that these thoughts aren't causing the actions. My body and brain were already gearing up to order a beer. The thought “I could kill for a beer” was just something that popped into my head as a result of what was happening in the physical brain and body. Thoughts don't cause actions.”*

“*For Epiphens, maybe,” replied Dirk*.

“*Well I can't see what's different about humans,” said Huxley, and for a while at least, nor could Dirk*.

(Baggini, J., The Pig That Wants to Be Eaten, 2005, p. 61.)

According to Baggini (2005) this fictional land of Epiphenia challenges the idea that no one can live with epiphenomenalism and where the crucial point is that “*how it feels to be an Epiphen is exactly the same as what feels like to be a human being. In both cases, thought accompanies action in just the same way. The only difference is that Epiphens do not believe their thoughts are doing any causing”* (p. 62).

While the compelling and self-evident explanation of the conscious-centric account does not undermine the logic of the epiphenomenalism argument, it is worth considering the epiphenomenalist's explanation for some everyday experiences.

When we experience pain for example, few consider that the excruciating hurt experienced is in fact a subjective representation generated by a biologically controlled, automatic protective hardwired system designed to signal corporeal damage or dysfunction with a view to encouraging protective/avoidance behaviors. Furthermore, most accept that the brain systems that generate the distinctive felt experience of pain can also provide sufficient cause for the range of related mental and behavioral responses to that pain (e.g., *crying, complaining, protecting the damaged body part)*. Accordingly, a consistent epiphenomenal view of pain would regard the neural brain processes as providing both the sufficient cause for both the mental state (i.e., felt painful experience) and associated behaviors previously attributed to the phenomenal state, whereas the psychological experience of the pain, while all too real, doesn't of itself cause anything.

Consequently, while both the phenomenal experience and the associated behaviors have underlying neural origins, their close temporal association supports a causal inference that persuades us that it was the feeling/experience that resulted in the behavior. In other words, the experience of what it is like to be in pain is not “something” with causal properties, but rather it is something generated by the brain and not a property of the experience *per se*. This is also true for other emotions- fear, anger, sadness, happiness, surprise, disgust, etc.

Likewise, when we verbalize what we are aware of when we think, the default inference is that what we say (the outputs) are *caused* by what we think. But an epiphenomenalist could equally argue that the thought and the verbalisations are both generated by underlying neural processes.

Consider another example by Baggini (2005) who explores the intuitive premise that appears to confirm the role of consciousness as the agent that lies behind the experience of thinking.

“*Let's say you're trying to work out a solution to a tricky logical or mathematical problem. Eventually, the eureka moment comes. In this case, surely the actual thinking has to play a part in the explanation for your actions?*

*Well, no. Why can't I believe that the conscious experience of thinking is just a by-product of the computing that is going on at brain level? It may be the necessary byproduct. But just as the noise that a boiling pot of water makes is an inevitable byproduct of the heating without that meaning it is the noise which cooks the egg, so thought could be the necessary by product of neural computation that doesn't itself produce the solution to the problem*.

*Indeed, if you think about thinking, there does seem to be something almost involuntary about it. Solutions “come to us”, for example, not we to them. Reflect on what it really feels like to think, and the idea that it is a byproduct of a process you are not conscious of may not seem quite so fanciful*” (p. 62).

The epiphenomenalist position does not deny subjective reality, but rather questions the natural, post-hoc causal inference linking thought and action. As argued in Halligan and Oakley (2018), the compelling evidence for the conscious centric account largely stems from the close and repeated temporal continuity of thoughts and actions (Blakemore et al., 2001;Woods et al., 2014). Given that “humans are causal determinists...” [we] “cannot help but experience the world as a continuous sequence of events and outcomes” (Hood, 2006). All our actions however have physical causes (i.e., neurophysiological changes in the brain), and therefore our intention, desire or volition to act, however intuitively compelling does not necessarily cause these very thoughts or related actions but rather are themselves caused by the physical brain processes that precede these thoughts and actions.

## Summary

We experience and explain ourselves at the level of our thoughts, decisions, and intentions. This self-evident (insider) view of consciousness with the executive agency to control our actions and psychological contents provides a compelling first person account that continues to shape scientific accounts of psychology and human behavior.

The distinction between first person (personal level) and third person (sub-personal levels) explanations for consciousness (originally described by Dennett (1969), offers two different explanatory frameworks of human behavior. Both provide different perspectives for how psychological processes are arranged. The primacy of the personal perspective inevitably influenced subsequent sub-personal accounts, ultimately fueling longstanding implicit dualism and support for conscious centric function and a functional distinction between conscious and non-conscious processes.

While we consider the intuitive explanation of consciousness to be a compelling social construct as real as free will, money, and equality, we suggest that the traditional conceptualization as an agentive consciousness is not supported by cognitive neuroscience and reflective introspections. Our account does not attempt to explain how the experience of being aware and mental states are generated by the brain, but rather seeks, in providing a scientific framework of our human psychology to dislodge the intuitive first person perspective of executive agency to that of a 3rd person perspective, where consciousness is regarded as an inert passive “personal narrative” that accompanies brain processes.

Although our ability to verbally report our subjective experiences will always remain an important variable as a readout of prior neurocognitive processes our account assumes no absolute, qualitative difference between conscious and non-conscious processing, preferring to see the traditional distinction between “automatic” and “controlled” cognitive processes as representing a qualitative indicator located along a continuum of neurocognitive awareness.

As noted previously, we consider the Personal Narrative (PN) to be, the product of non-conscious neurocognitive activity located in the Central Executive Structure internally broadcasting selective processes and cognitive products related to the “task in hand”.

The adaptive evolutionary advantage of the PN resides in the capacity for a selection of psychological contents to be communicated to others via language, gestures, physical artfacts, etc. - contributing ultimately to the evolution of cultural resources. The content of the PN is also selectively transferred to episodic memory as a resource.

Although epiphenomenalism will always remain, personally and emotionally a deeply unsatisfying position this conceptual repositioning provides a framework that relocates non-conscious neurocognitive processes to the center of any scientific endeavor to explain how the human brain is psychologically organized (the machinery) rather than focusing on the subjective experience (the ghost).

## Author Contributions

All authors listed have made a substantial, direct and intellectual contribution to the work, and approved it for publication.

## Conflict of Interest

The authors declare that the research was conducted in the absence of any commercial or financial relationships that could be construed as a potential conflict of interest.
